# Supplementary material for: Benchmarking tools for detecting longitudinal differential expression in proteomics data allows establishing a robust reproducibility optimization regression approach
Source: Nat Commun. 2022 Dec 22;13:7877. doi: 10.1038/s41467-022-35564-z (PMC9780321; doi:10.1038/s41467-022-35564-z)
Supplement: Supplementary file 1 — Supplementary Information [file 41467_2022_35564_MOESM1_ESM.pdf]

Benchmarking tools for detecting longitudinal differential expression in proteomics data allows establishing a robust reproducibility optimization regression approach

Tommi Välikangas, Tomi Suomi, Courtney E. Chandler, Alison J Scott, Bao Q. Tran, Robert K. Ernst, David R. Goodlett, Laura L. Elo

Supplementary Information

Supplementary Note

Robustness of the new method RoIDE

While the default usage of RoIDE is very easy, the user is allowed full control of all the parameters, including the polynomial degree in the RegROTS and PolyReg modules, the use of random effects for the individual baseline or slope in the PolyReg module, and the number of bootstraps in the RegROTS and DiffROTS modules. To evaluate the sensitivity of RoIDE to these parameters, we explored how the different degrees for the RegROTS and PolyReg modules and the use of a random effect for the individual baseline in the PolyReg module affected the performance of RoIDE in the semi-simulated spike-in datasets (Supplementary Figure 5). Regardless of the used degrees for RegROTS and PolyReg or the model type for the PolyReg module, only slight variations were observed and the performance of RoIDE remained excellent despite the specific parameters used. Importantly, using the default settings, RoIDE automatically selected the best performing parameter combination, supporting the utility of the automatic parameter selection approach (Supplementary Figure 5). Similarly, increasing the number of bootstraps beyond the default value of 100 for the RegROTS and DiffROTS modules did not have a major effect on the performance of the method.

To provide a more refined evaluation of the RoIDE methodology, we assessed in more detail the effect of the reproducibility-optimization with ROTS as well as the combination of multiple rankings on the performance. First, to demonstrate the benefits of ROTS, we composed a variant of the RegROTS module utilizing a standard one-way Analysis of Variance (ANOVA) instead of ROTS and extensively compared this RegANOVA method to the RegROTS module in the 1920 semi-simulated datasets. The comparisons clearly demonstrated the benefits of applying ROTS over ANOVA with considerable performance gains ($p < 10^{-6}$ in all scenarios, Supplementary Figure 8).

Although already the RegROTS module alone performed very well, the use of all the complementary modules (RegROTS, DiffROTS and PolyReg) increased the performance further (Supplementary Figure 8). Ultimately, the combination of the diverse approaches through rank products stabilizes the composite method and allows for consistent excellent performance in diverse datasets. To further investigate the effect of combining multiple approaches, we also combined three established diverse methods Timecourse, Limma and MaSigPro using a similar ranking and rank product approach as in RoIDE in two different types of datasets, the semi-simulated SGSDS full and the UPS1 Mix full datasets (Supplementary Figure 9). Indeed, the composite approach of Timecourse, Limma and MaSigPro performed relatively well in both types of dataset. However, in the UPS1 Mix datasets, the performance was significantly reduced, when compared to Timecourse alone ($p < 10^{-15}$, Supplementary Figure 9). This demonstrates how the combination of multiple approaches does not always improve the results but can also reduce the performance, highlighting the importance of careful consideration regarding such combination.

Finally, to comprehensively evaluate the simulation-based estimation of the significance values and the false discovery rate control of RoIDE, we explored the numbers of false discoveries in 600 varying datasets under the null hypothesis. For this purpose, 200 completely random (noise) datasets were generated similarly as the UPS1-based semi-simulated datasets but with random draws of all values from a same normal distribution. In addition to such completely random datasets, 200 protein-wise random datasets were generated using protein-wise normal distributions. Thus, each protein had a distinct protein specific mean and standard deviation, following those from the corresponding semi-simulated spike-in dataset based on real experimental data. As both the completely random datasets and the protein-wise datasets were generated randomly, they did not contain any clear patterns. Therefore, we also generated datasets where there were clear patterns for some proteins in the data, but this pattern did not differ between the two conditions. For this, 200 UPS1-based semi-

simulated datasets were randomly selected and only the samples from one condition were used, while the other condition was generated by replicating the samples from the first condition with random noise from normal distribution. In each dataset type, datasets with no missing values, and with 5%, 10% and 15% of missing values were generated. Our results demonstrate the ability of the simulation approach to effectively estimate the significance values and control the number of false discoveries (Supplementary Table 3). In the absence of true longitudinal differential expression signal in the data, false detections were very rare, confirming the effectiveness of RolDE in controlling false discoveries.

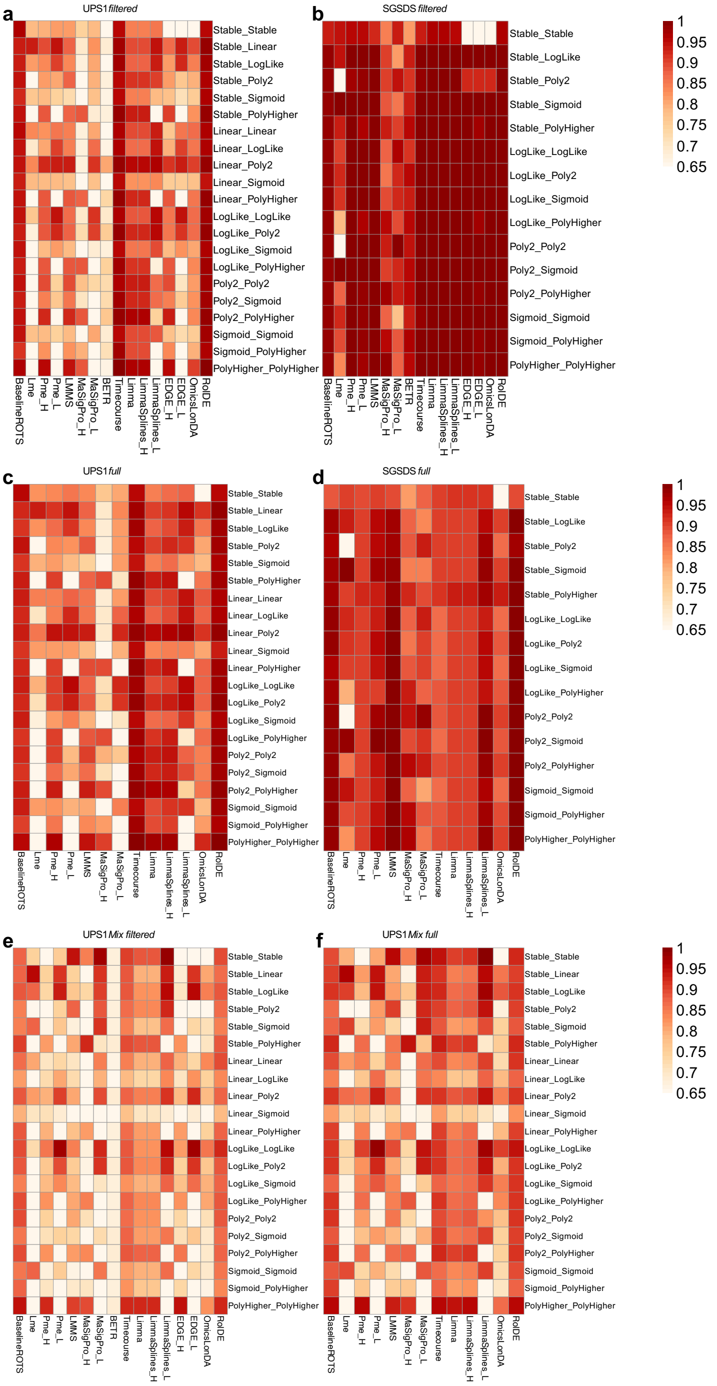

Supplementary Figure 1. Performance of the different methods (columns) across the trend difference categories (rows) in the semi-simulated spike-in datasets. **(a)** UPS1 filtered (n=300 datasets), **(b)** SGSDS filtered (n=210 datasets), **(c)** UPS1 full (n=300 datasets), **(d)** SGSDS full (n=210 datasets), **(e)** UPS1 Mix filtered (n=300 datasets), **(f)** UPS1 Mix full (n=300 datasets). The methods were examined in their ability to detect true known longitudinal differential expression using receiver operating characteristic (ROC) analysis across datasets with varying longitudinal trend differences in the spike-in proteins (3 replicate samples per condition). The partial areas under the ROC curves (pAUC) between the specificity of 1 and 0.9 were used to measure the performance of the methods. The interquartile range (IQR) mean pAUCs are presented with the colour scale. Source data are provided as a Source Data file.

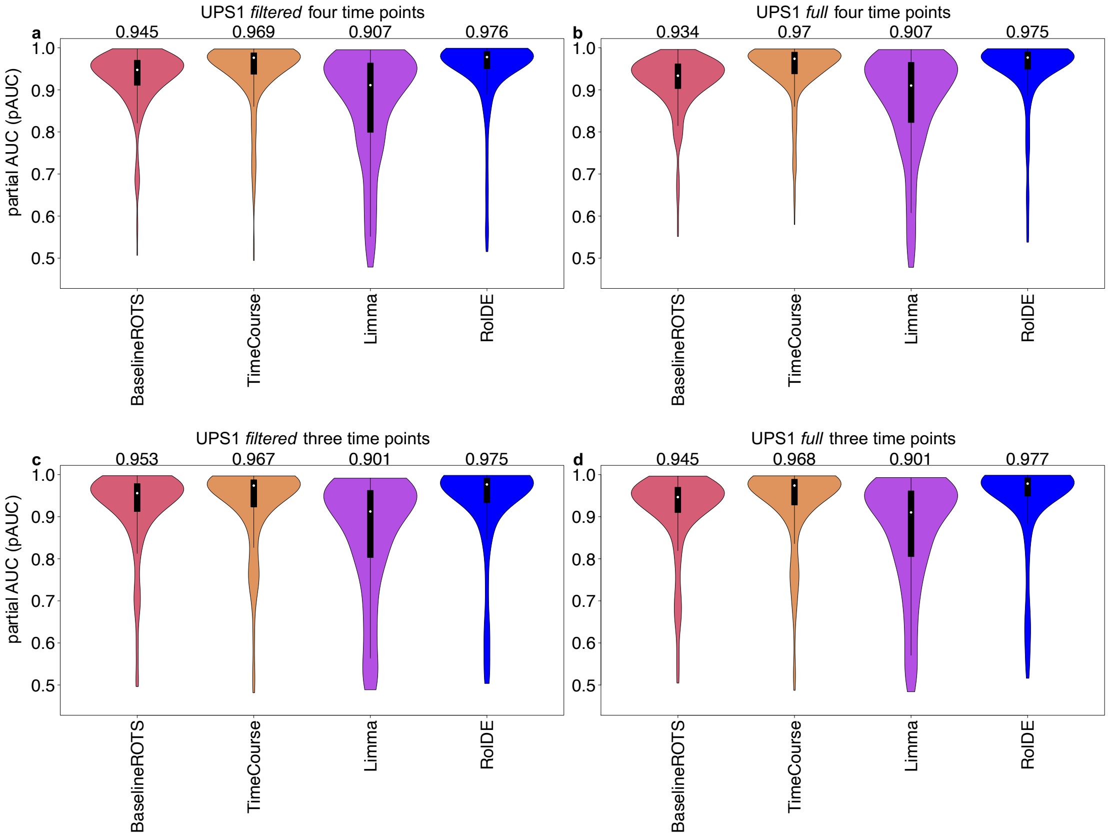

Supplementary Figure 2. Performance of the selected best-performing methods RolDE, Timecourse, Limma, and the baseline ROTS in semi-simulated spike-in datasets with only three or four time points. **(a)** UPS1 filtered with four time points (n=300 datasets), **(b)** UPS1 full with four time points (n=300 datasets), **(c)** UPS1 filtered with three time points (n=300 datasets), **(d)** UPS1 full with three time points (n=300 datasets). The methods were examined in their ability to detect true known longitudinal differential expression using receiver operating characteristic (ROC) analysis across datasets with varying longitudinal trend differences in the spike-in proteins (3 replicate samples per condition). The partial areas under the ROC curves (pAUC) between the specificity of 1 and 0.9 were used to measure the performance of the methods. The violin plots display the distribution of pAUCs for each method, including median (white circle), interquartile range (IQR) from the first to third quartile (black box), and 1.5* IQR (whiskers). The IQR mean pAUC for each method is shown above the violin. Each method is shown with a unique colour. Source data are provided as a Source Data file.

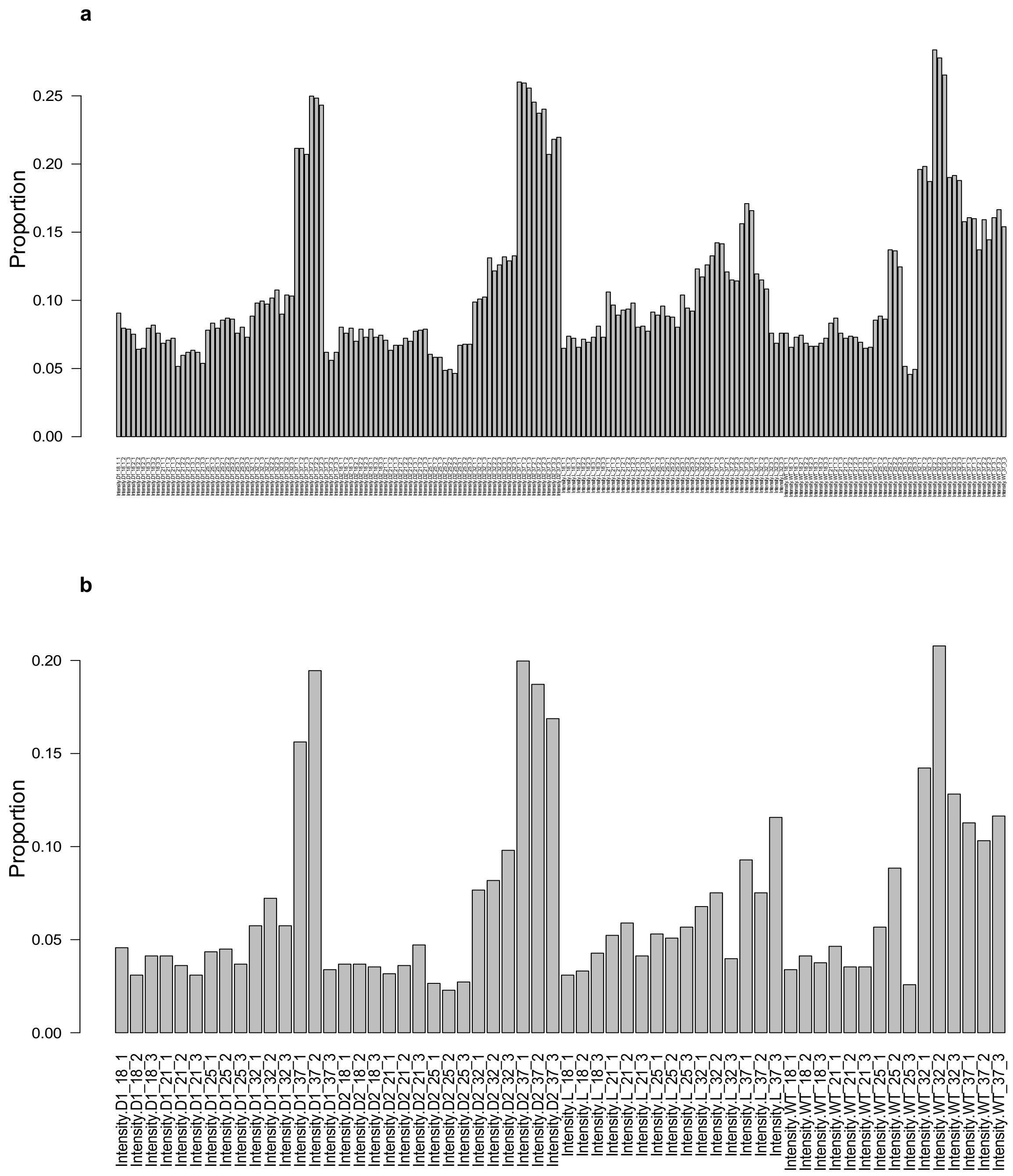

Supplementary Figure 3. Missing value proportions in the samples of the *Francisella tularensis* subspecies *novicida* proteomics dataset. Missing value proportions in **(a)** all the samples, and **(b)** after averaging over the technical replicates for a biological replicate. Source data are provided as a Source Data file.

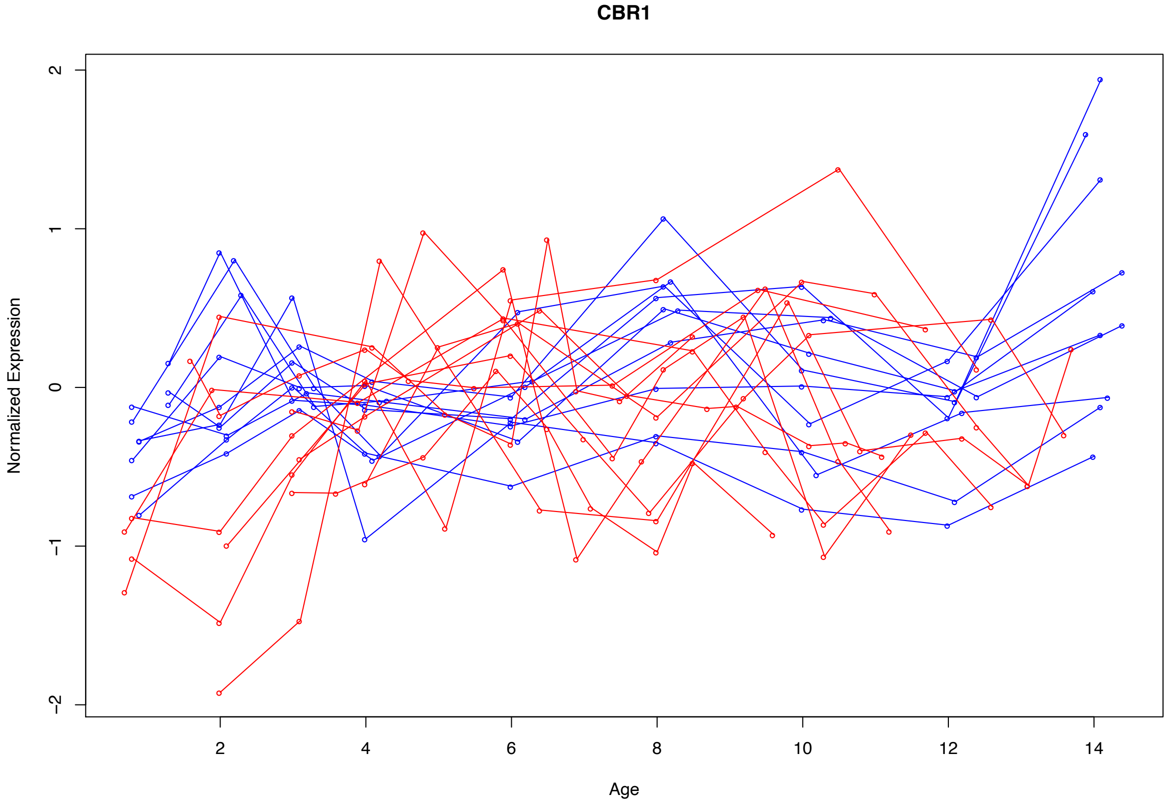

Supplementary Figure 4. A significant finding (FDR = 0.04) by the original study by Liu et al. in their differential expression analysis of the longitudinal blood plasma proteome of 11 children developing type 1 diabetes (red lines) and 10 matched controls (blue lines), carbonyl reductase 1 (CBR1). With RolDE, CBR1 had FDR of 0.07 for longitudinal differential expression. Source data are provided as a Source Data file.

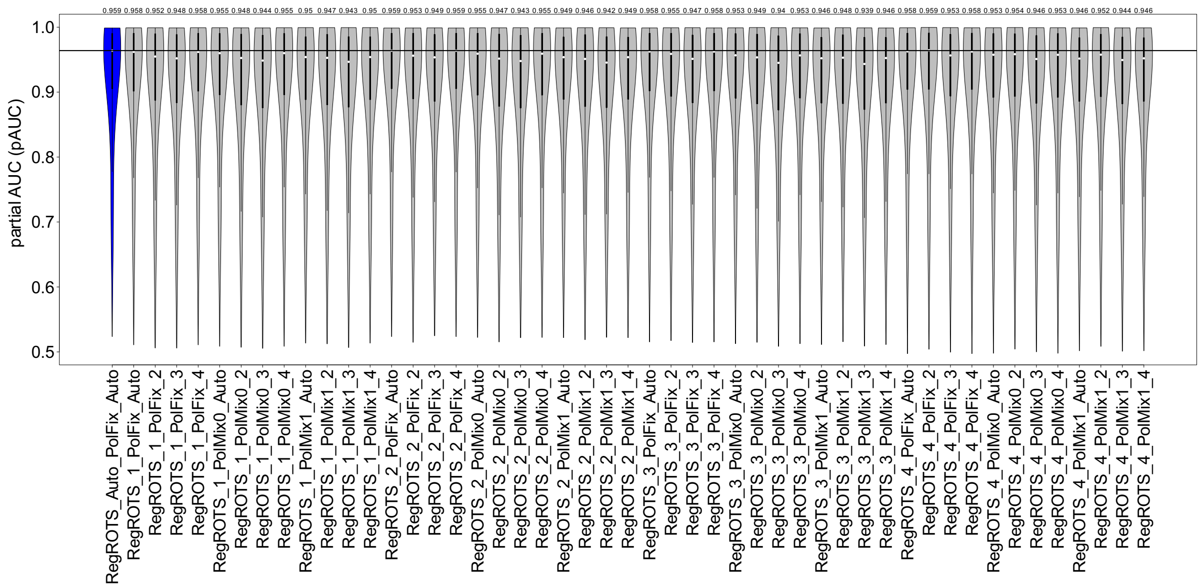

Supplementary Figure 5. Performance of the proposed new method RolDE using different parameters for its modules. The polynomial degrees in the RegROTS and PolyReg modules were varied from 1 to 4. In addition, the model type for the PolyReg module was varied, including only fixed models (PolFix), mixed models with a random effect for the individual baseline (PolMix0), and mixed models with random effects for the individual baseline and slope (PolMix1). The performance of RolDE with the different parameters was examined over 1920 semi-simulated spike-in datasets (300 UPS1 filtered, 300 UPS1 full, 300 UPS1 Mix filtered, 300 UPS1 Mix full, 210 SGSDS filtered, 210 SGSDS full, and 300 CPTAC full datasets) using receiver operating characteristic (ROC) analysis with varying longitudinal trend differences between the conditions in the spike-in proteins (3 replicate samples per condition). The partial areas under the ROC curves (pAUC) between the specificity of 1 and 0.9 were used to measure the performance of the parameter settings. The violin plots display the distribution of pAUCs for each setting, including median (white circle), interquartile range (IQR) from the first to third quartile (black box), and 1.5* IQR (whiskers). The IQR mean pAUCs are shown above the violins. The horizontal black line represents the IQR mean pAUC of the default approach of RolDE that determines the degrees automatically. Source data are provided as a Source Data file.

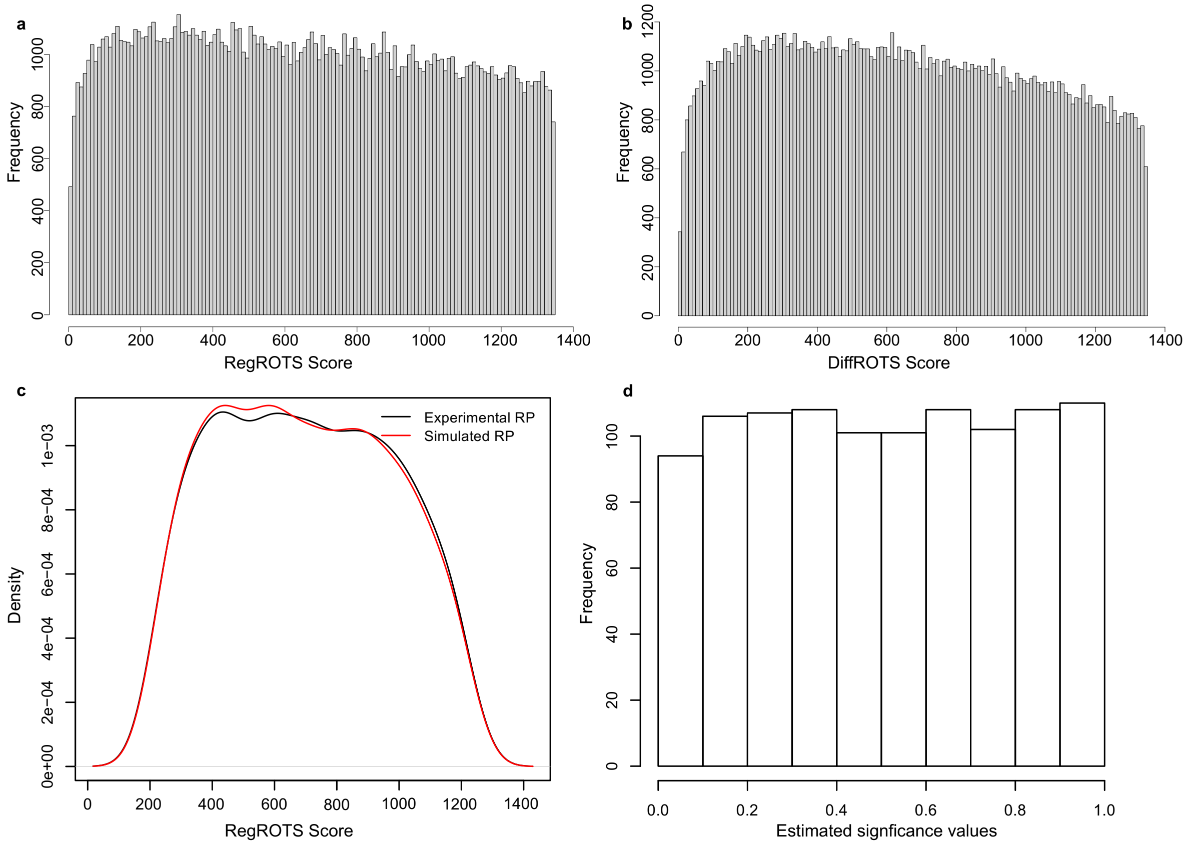

Supplementary Figure 6. Empirical distributions of specific RolDE modules under the null hypothesis and example of significance value estimation in RolDE. **(a)** The empirical distribution of the RegROTS scores $S_{RegROTS}$ under the null hypothesis. **(b)** The empirical distribution of the DiffROTS scores $S_{DiffROTS}$ under the null hypothesis. To determine the empirical distributions, completely random (noise) datasets were generated on the basis of the UPS1 semi-simulated datasets with random draws of the values from a normal distribution. **(c)** Distribution of the experimental and simulated internal rank products for the RegROTS module in a simulated random longitudinal data and **(d)** the corresponding estimated significance values for RolDE in the same data. Similar to the UPS1-based datasets, the random dataset contained two conditions, five time points, and three replicates in each condition.

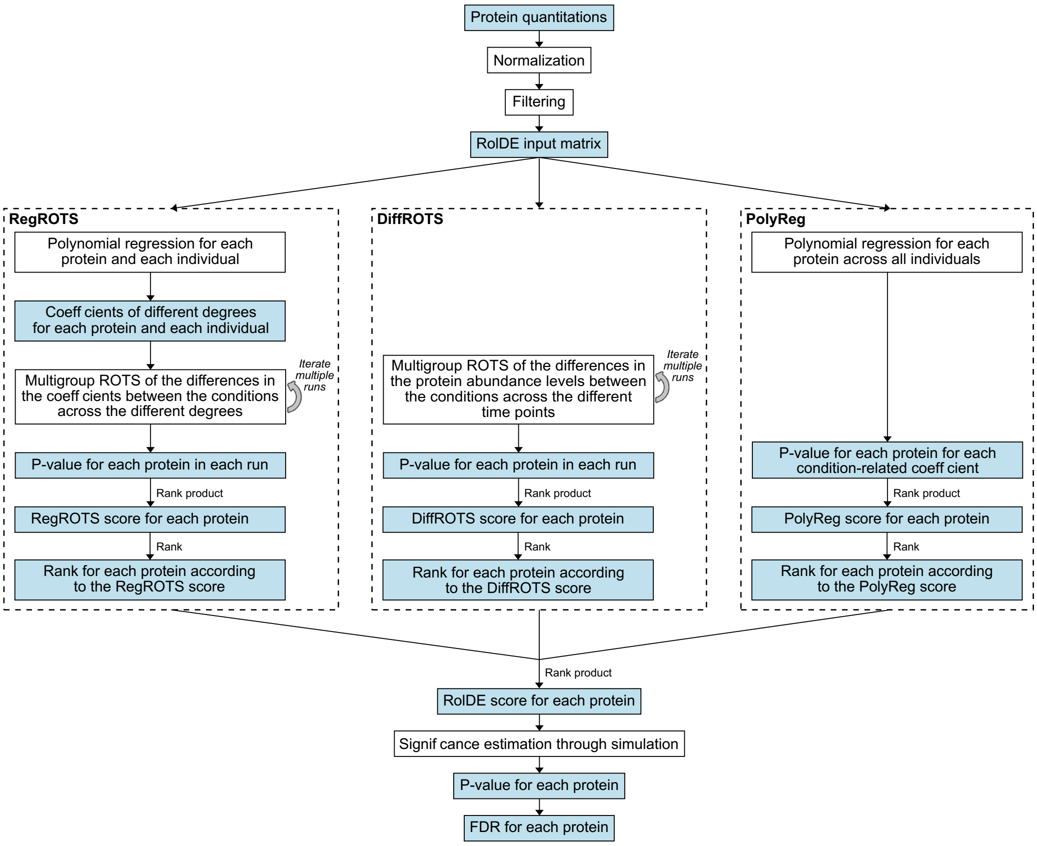

Supplementary Figure 7. The RolDE workflow. In case of non-aligned time points, the DiffROTS module is slightly adjusted.

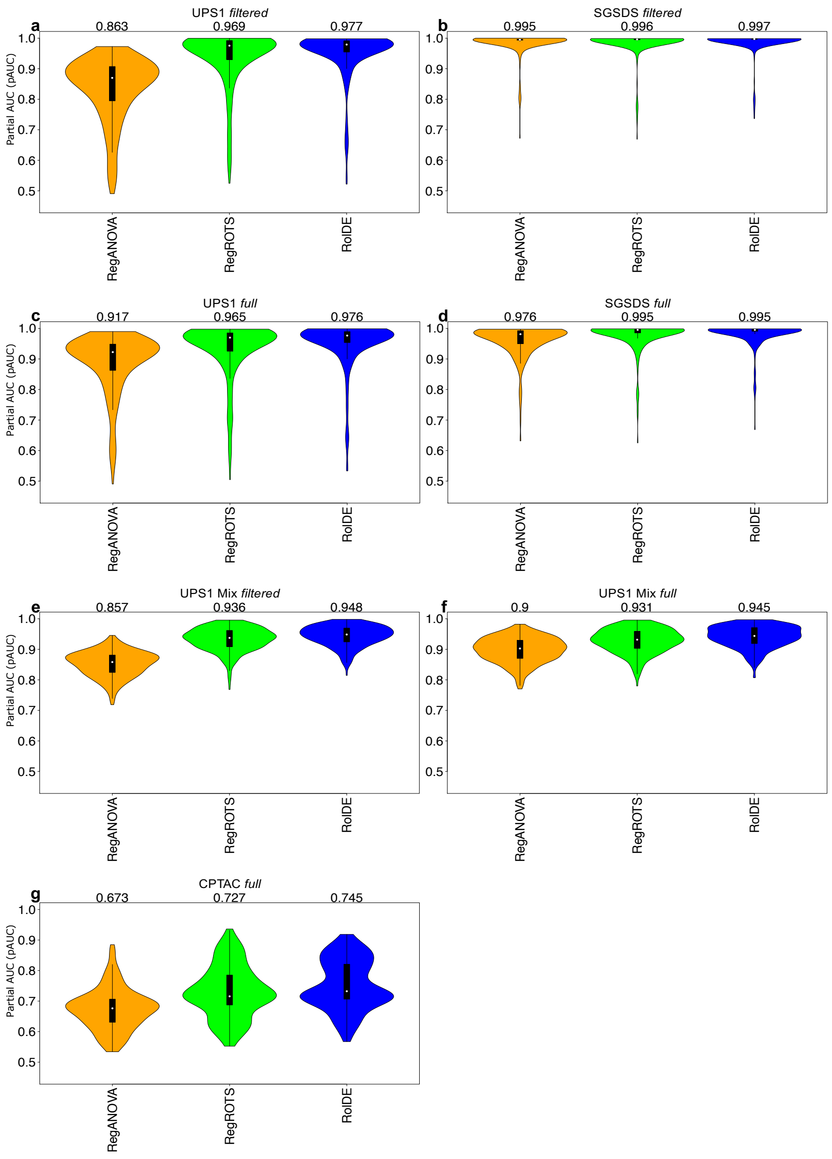

Supplementary Figure 8. Performance of the RegROTS module, the RegANOVA approach, and the proposed new method RoIDE in the semi-simulated spike-in datasets. **(a)** UPS1 filtered (n=300 datasets), **(b)** SGSDS filtered (n=210 datasets), **(c)** UPS1 full (n=300 datasets), **(d)** SGSDS full (n=210 datasets), **(e)** UPS1 Mix filtered (n=300 datasets), **(f)** UPS1 Mix full (n=300 datasets), (**g**) CPTAC full (n=300 datasets). The methods were examined in their ability to detect true known longitudinal differential expression using receiver operating characteristic (ROC) analysis across datasets with varying longitudinal trend differences in the spike-in proteins (3 replicate samples per condition). The partial areas under the ROC curves (pAUC) between the specificity of 1 and 0.9 were used to measure the performance of the methods. The violin plots display the distribution of pAUCs for each method, including median (white circle), interquartile range (IQR) from the first to third quartile (black box), and 1.5* IQR (whiskers). The IQR mean pAUC for each method is shown above the violin. Each method is shown with a unique colour. In the RegANOVA approach, the Reproducibility Optimized Test Statistic (ROTS) was replaced with the standard One-Way Analysis of Variance (ANOVA) approach to evaluate the benefits of the ROTS approach. Source data are provided as a Source Data file. Source data are provided as a Source Data file.

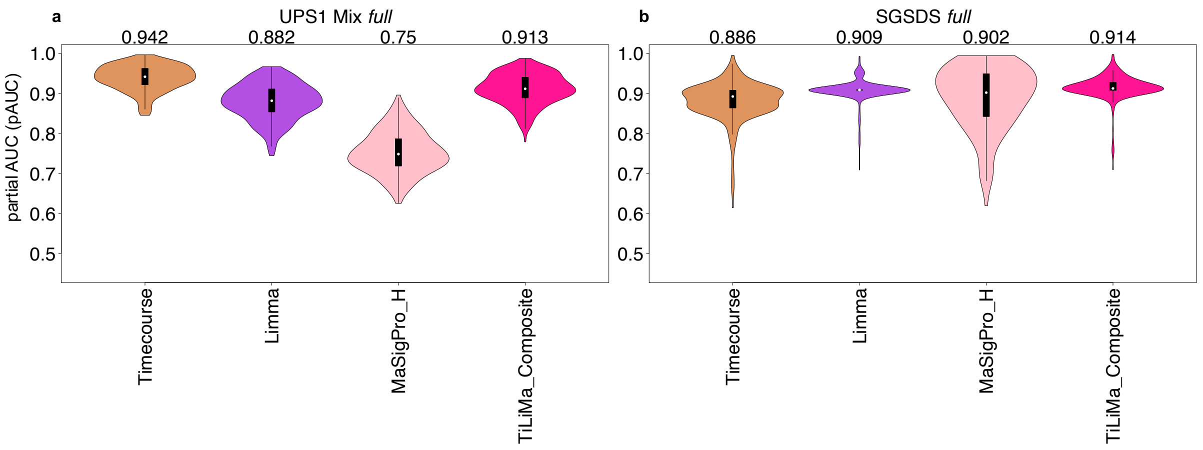

Supplementary Figure 9. Performance of Timecourse, Limma and MaSigPro and their composite approach in the semi-simulated spike-in datasets. (**a)** UPS1 Mix full (n=300 datasets), **(b)** SGSDS full (n=210 datasets). The methods were examined in their ability to detect true known longitudinal differential expression using receiver operating characteristic (ROC) analysis across datasets with varying longitudinal trend differences in the spike-in proteins (3 replicate samples per condition). The partial areas under the ROC curves (pAUC) between the specificity of 1 and 0.9 were used to measure the performance of the methods. The violin plots display the distribution of pAUCs for each method, including median (white circle), interquartile range (IQR) from the first to third quartile (black box), and 1.5* IQR (whiskers). The IQR mean pAUC for each method is shown above the violin. Each method is shown with a unique colour. The three methods were combined using rank product similarly as in the RolDE approach. Source data are provided as a Source Data file.

Supplementary Table 1. All unique proteins in the KEGG Lipopolysaccharide synthesis pathway (ftn00540) complemented with unique proteins from the associated Lipopolysaccharide biosynthesis knockout pathway (ko00540). Proteins belonging to the KEGG Lipopolysaccharide synthesis pathway (ftn00540) are highlighted. The included pathway proteins used for the gene set enrichment analysis (GSEA) in different comparisons are shown in columns.

Entry	Entry_name	Status	Gene_names	Gene_names_primary	Included in GSEA, WT vs. L comparison	Included in GSEA, WT vs. D2 comparison	Included in GSEA, WT vs. D1 comparison
A0Q7Y0	A0Q7Y0_FRATN	unreviewed	lpxA FTN_1478	lpxA			x
A0Q4B0	A0Q4B0_FRATN	unreviewed	lpxC FTN_0165	lpxC			
A0Q7Y2	A0Q7Y2_FRATN	unreviewed	lpxD FTN_1480	lpxD			x
A0Q4E5	A0Q4E5_FRATN	unreviewed	lpxD FTN_0200	lpxD		x	x
A0Q5A8	A0Q5A8_FRATN	unreviewed	lpxH FTN_0528	lpxH	x	x	x
A0Q7X9	LPXB_FRATN	reviewed	lpxB FTN_1477	lpxB			
A0Q8A0	LPXK_FRATN	reviewed	lpxK FTN_1605	lpxK		x	
A0Q788	A0Q788_FRATN	unreviewed	kpsF FTN_1222	kpsF	x	x	x
A0Q5J1	KDSA_FRATN	reviewed	kdsA FTN_0611	kdsA	x	x	x
A0Q6C8	A0Q6C8_FRATN	unreviewed	yrbI FTN_0905	yrbI		x	
A0Q5R0	KDSB_FRATN	reviewed	kdsB FTN_0683	kdsB	x	x	x
A0Q7X2	A0Q7X2_FRATN	unreviewed	kdtA FTN_1469	kdtA			
A0Q418	A0Q418_FRATN	unreviewed	FTN_0072	#N/A			
A0Q417	A0Q417_FRATN	unreviewed	FTN_0071	#N/A	x		
A0Q450	A0Q450_FRATN	unreviewed	FTN_0104	#N/A			
A0Q504	A0Q504_FRATN	unreviewed	lpxE FTN_0416	lpxE	x	x	x
A0Q576	A0Q576_FRATN	unreviewed	kdoH1 FTN_0495	kdoH1		x	
A0Q4N6	LPXF_FRATN	reviewed	lpxF FTN_0295 AW25_1746	lpxF	x	x	x

Supplementary Table 2. The detected longitudinally differentially expressed proteins at false discovery rate (FDR) of 0.05 using the Robust longitudinal Differential Expression method RoIDE in the longitudinal type 1 diabetes blood plasma proteomics data of Liu et al.

Feature ID	RoIDE Rank Product	Estimated false discovery rate
TRFE (P02787)	15.4	$<10^{-16}$
SCLT1 (Q96NL6)	18.6	$<10^{-16}$
CGRE1 (Q99674)	30.0	$<10^{-16}$
K1H1 (Q15323)	38.0	$<10^{-16}$
SAA1 (P0DJI8)	42.5	$<10^{-16}$
A0A0G2JH38	53.4	0.0006
TSK (Q8WUA8)	55.5	0.0006
AMYP (P04746)	59.8	0.0008
LY66F (Q5SQ64)	60.8	0.0008
KRT86 (O43790)	63.9	0.0008
RAB2A (P61019)	64.6	0.0008
PA1B3 (Q15102)	72.7	0.0017
ASAP1 (Q9ULH1)	75.8	0.0035
FAT4 (Q6V0I7)	80.8	0.0057
CISY (O75390)	86.5	0.0279

Supplementary Table 3. False discovery rate (FDR) with RolDE in various types of datasets under the null hypothesis, including 200 completely random (noise) datasets, 200 protein-wise random datasets, and 200 datasets with clear patterns for some proteins but without differences between the two conditions. For each dataset type, datasets with no missing values, and datasets with 5%, 10% and 15% of missing values were generated. Median and range are shown.

Dataset Type (under H0)	FDR range	FDR median
Completely random - no missing values	0 - 0.001	0
Completely random - 5% missing values	0 - 0.001	0
Completely random - 10% missing values	0 - 0.001	0
Completely random - 15% missing values	0 - 0	0
Proteinwise random - no missing values	0 - 0.0029	0.0009
Proteinwise random - 5% missing values	0 - 0.0019	0
Proteinwise random - 10% missing values	0 - 0.001	0
Proteinwise random - 15% missing values	0 - 0.001	0
One condition random - no missing values	0 - 0.001	0
One condition random - 5% missing values	0 - 0	0
One condition random - 10% missing values	0 - 0	0
One condition random - 15% missing values	0 - 0	0
