# Supplementary material for: Benchmarking tools for detecting longitudinal differential expression in proteomics data allows establishing a robust reproducibility optimization regression approach
Source: Nat Commun. 2022 Dec 22;13:7877. doi: 10.1038/s41467-022-35564-z (PMC9780321; doi:10.1038/s41467-022-35564-z)
Supplement: Supplementary file 3 — Description of Additional Supplementary Files [file 41467_2022_35564_MOESM3_ESM.docx]

**File name: Supplementary Data 1.**

**Description:** All the generated trends and their combinations in the semi-simulated datasets based on the UPS1, SGSDS and CPTAC spike-in datasets with a single trend (SemiSimulated_Spike_In_Data_SampleGroups.xlsx) or with varying trends within a condition (SemiSimulated_Mix_Datasets_SampleGroups.xlsx), as well as visualization of the trends (SemiSimulated_Spike_In_Data_Trends.pdf).

**File name: Supplementary Data 2.**

**Description:** All the gene sets used for the gene set enrichment analysis in the human induced T regulatory cell (iTreg) data.
