# Supplementary material for: Benchmarking tools for detecting longitudinal differential expression in proteomics data allows establishing a robust reproducibility optimization regression approach
Source: Nat Commun. 2022 Dec 22;13:7877. doi: 10.1038/s41467-022-35564-z (PMC9780321; doi:10.1038/s41467-022-35564-z)
Supplement: Supplementary file 6 — Reporting Summary [file 41467_2022_35564_MOESM6_ESM.pdf]

nature portfolio

Corresponding author(s): Laura Elo

Last updated by author(s): Dec 6, 2022

Reporting Summary

Nature Portfolio wishes to improve the reproducibility of the work that we publish. This form provides structure for consistency and transparency in reporting. For further information on Nature Portfolio policies, see our Editorial Policies and the Editorial Policy Checklist.

Statistics

For all statistical analyses, confirm that the following items are present in the figure legend, table legend, main text, or Methods section.

n/a	Confirmed	
☐	☒	The exact sample size (*n*) for each experimental group/condition, given as a discrete number and unit of measurement
☐	☒	A statement on whether measurements were taken from distinct samples or whether the same sample was measured repeatedly
☐	☒	The statistical test(s) used AND whether they are one- or two-sided<br>*Only common tests should be described solely by name; describe more complex techniques in the Methods section.*
☐	☒	A description of all covariates tested
☐	☒	A description of any assumptions or corrections, such as tests of normality and adjustment for multiple comparisons
☐	☒	A full description of the statistical parameters including central tendency (e.g. means) or other basic estimates (e.g. regression coefficient) AND variation (e.g. standard deviation) or associated estimates of uncertainty (e.g. confidence intervals)
☐	☒	For null hypothesis testing, the test statistic (e.g. *F*, *t*, *r*) with confidence intervals, effect sizes, degrees of freedom and *P* value noted<br>*Give P values as exact values whenever suitable.*
☒	☐	For Bayesian analysis, information on the choice of priors and Markov chain Monte Carlo settings
☒	☐	For hierarchical and complex designs, identification of the appropriate level for tests and full reporting of outcomes
☐	☒	Estimates of effect sizes (e.g. Cohen's *d*, Pearson's *r*), indicating how they were calculated

Our web collection on statistics for biologists contains articles on many of the points above.

Software and code

Policy information about availability of computer code

Data collection	Publicly available datasets were directly downloaded from their sources.
Data analysis	Raw mass spectrometry data were processed using MaxQuant 1.5.3.30 (spike-in datasets) or 1.6.5.0 (Fn data). Data analysis was performed using R statistical programming software 3.6.1 or 4.00 (semi-simulated datasets) or 4.0.5 (Fn, iTreg and T1D data) along with the packages: RolDE developer version 0.99.4, ROTS 1.12.0 (semi-simulated datasets) or 1.18.0 (Fn, iTreg and T1D data), betr 1.32.0, limma 3.40.2-3.40.6 (semi-simulated datasets) or 3.46.0 (Fn, iTreg and T1D data), timecourse 1.56.0 (semi-simulated datasets) or 1.62.0 (Fn, iTreg and T1D data), maSigPro 1.56.0 (semi-simulated datasets) or 1.62.0 (Fn, iTreg and T1D data), lmms 1.3.3, edge 2.16.0 (semi-simulated datasets) or 2.22.0 (Fn, iTreg and T1D data), OmicsLonDA 1.6.0, nlme 3.1-142-3.1-149 (semi-simulated datasets) or 3.1-152 (Fn, iTreg and T1D data), fgsea 1.10.1-1.16.0, pROC 1.15.3-1.18.0, vioplot 0.3.2-0.3.7, pheatmap 1.0.12, venn 1.10. Custom codes for the methods applied in the study are available in GitHub at https://github.com/elolab/RolDE-benchmarking.

For manuscripts utilizing custom algorithms or software that are central to the research but not yet described in published literature, software must be made available to editors and reviewers. We strongly encourage code deposition in a community repository (e.g. GitHub). See the Nature Portfolio guidelines for submitting code & software for further information.

Data

Policy information about availability of data

All manuscripts must include a data availability statement. This statement should provide the following information, where applicable:

- Accession codes, unique identifiers, or web links for publicly available datasets
- A description of any restrictions on data availability
- For clinical datasets or third party data, please ensure that the statement adheres to our policy

The UPS1 spike-in dataset is available from PRIDE with the identifier PXD002099 [https://www.ebi.ac.uk/pride/archive/projects/PXD002099]. The SGSDS spike-in dataset is available from PeptideAtlas: No. PASS00589 [http://www.peptideatlas.org/PASS/PASS00589] (username PASS00589, password WF6554orn). The CPTAC spike-in dataset (study 6, at test site 86) is available from the CPTAC Portal [https://cptac-data-portal.georgetown.edu/cptac/study/list?scope=Phase+I]. The Francisella tularensis subspecies novicida (Fn) data generated in this study has been deposited in the ProteomeXchange Consortium via the PRIDE partner repository under accession code PXD025439 [https://www.ebi.ac.uk/pride/archive/projects/PXD025439]. The type 1 diabetes dataset of Liu et al. is available from the original publication (Supplementary Tables S1-S3 of the original publication). The human iTreg dataset of Schmidt et al. is available from the original publication (Supplementary Table S2 of the original publication). The gene sets for the enrichment analysis were downloaded from the the Molecular Signatures Database MSigDB (v2022.1) [https://www.gsea-msigdb.org/gsea/msigdb], Reactome (version 81) [https://reactome.org], the STRING database (11.5) [https://string-db.org], and the original publication of Ferraro et al. (Supplementary Dataset S2 of the original publication).

Field-specific reporting

Please select the one below that is the best fit for your research. If you are not sure, read the appropriate sections before making your selection.

☒ Life sciences ☐ Behavioural & social sciences ☐ Ecological, evolutionary & environmental sciences

For a reference copy of the document with all sections, see nature.com/documents/nr-reporting-summary-flat.pdf

Life sciences study design

All studies must disclose on these points even when the disclosure is negative.

Sample size	Sample sizes for the semi-simulated spike-in datasets were determined according to the original published datasets. Sample sizes for the previously published type 1 diabetes and iTreg datasets were determined in the original studies. For the Francisella tularensis subspecies novicida (Fn) dataset, 3 independent biological replicates were used in each strain and temperature, with 3 technical replicates for each, which was expected to be sufficient to examine longitudinal differential protein expression based on previous experience. No specific sample-size calculation was performed.
Data exclusions	No data was excluded from the study.
Replication	The methods were evaluated and compared using over 3000 semi-simulated and three large experimental datasets to robustly assess the performance of different methods in detecting differentially abundant proteins longitudinally.
Randomization	Randomization was not relevant as the study involved re-analysis of published datasets, analysis of semi-simulated datasets, and analysis of laboratory strains of Francisella tularensis.
Blinding	Blinding was not relevant as the study involved re-analysis of published datasets and the semi-simulated and Fn datasets did not contain subjective measurements. However, all the software tools were computationally run and assessed with as little human interference as possible, using a priori determined assessment criteria.

Reporting for specific materials, systems and methods

We require information from authors about some types of materials, experimental systems and methods used in many studies. Here, indicate whether each material, system or method listed is relevant to your study. If you are not sure if a list item applies to your research, read the appropriate section before selecting a response.

Materials & experimental systems

n/a	Involved in the study	
☒	☐	Antibodies
☒	☐	Eukaryotic cell lines
☒	☐	Palaeontology and archaeology
☒	☐	Animals and other organisms
☒	☐	Human research participants
☒	☐	Clinical data
☒	☐	Dual use research of concern

Methods

n/a	Involved in the study	
☒	☐	ChIP-seq
☒	☐	Flow cytometry
☒	☐	MRI-based neuroimaging
